# Involvement of methylation of MicroRNA-122, −125b and -106b in regulation of Cyclin G1, CAT-1 and STAT3 target genes in isoniazid-induced liver injury

**DOI:** 10.1186/s40360-018-0201-x

**Published:** 2018-03-20

**Authors:** Yuhong Li, Qi Ren, Lingyan Zhu, Yingshu Li, Jinfeng Li, Yiyang Zhang, Guoying Zheng, Tiesheng Han, Shufeng Sun, Fumin Feng

**Affiliations:** 1 Hebei Province Key Laboratory of Occupational Health and Safety for Coal Industry, School of Public Health, North China University of Science and Technology, No.21 Bohai Road, Tangshan, 063210 People’s Republic of China; 2 College of Nursing and Rehabilitation, North China University of Science and Technology, Tangshan, 063210 China

## Abstract

**Background:**

This investigation aimed to evaluate the role of methylation in the regulation of microRNA (miR)-122, miR-125b and miR-106b gene expression and the expression of their target genes during isoniazid (INH)-induced liver injury.

**Methods:**

Rats were given INH 50 mg kg^− 1^·d^− 1^ once per day for 3, 7, 10, 14, 21 and 28 days and were sacrificed. Samples of blood and liver were obtained.

**Results:**

We analysed the methylation and expression levels of miR-122, miR-125b and miR-106b and their potential gene targets in livers. Liver tissue pathologies, histological scores and alanine aminotransferase (ALT) and aspartate aminotransferase (AST) activities changed, indicating the occurrence of liver injury. Relative expression levels of miR-122, miR-125b and miR-106b genes in the liver decreased after INH administration and correlated with the scores of liver pathology and serum AST and ALT activities, suggesting that miR-122, miR-125b and miR-106b are associated with INH-induced liver injury. The amount of methylated miR-122, miR-125b and miR-106b in the liver increased after INH administration and correlated with their expression levels, suggesting the role of methylation in regulating miRNA gene expression. Two miR-122 gene targets, cell cycle protein G1 (Cyclin G1) and cationic amino acid transporter-1 (CAT-1), also increased at the mRNA and protein levels, which suggests that lower levels of miR-122 contribute to the upregulation of Cyclin G1 and CAT-1 and might play a role in INH-induced liver injury. Signal transducer and activator of transcription 3 (STAT3) was a common target gene of miR-125b and miR-106b, and its expression levels of mRNA and protein increased after INH administration. The protein expression of phosphorylated (p)-STAT3 and the mRNA expression of RAR-related orphan receptor gamma (RORγt) regulated by p-STAT3 also increased. Meanwhile, the mRNA and protein expression of interleukin (IL)-17 regulated by RORγt, and the mRNA and protein expression of CXCL1 and MIP-2 regulated by IL-17 increased after INH administration. These results demonstrate that lower levels of hepatic miR-125b and miR-106b contribute to the upregulation of STAT3 in stimulating the secretion of inflammatory factors during INH-induced liver injury.

**Conclusions:**

Our results suggested that DNA methylation probably regulates the expression of miRNA genes (miR-122, miR-125b, and miR-106b), affecting the expression of their gene targets (Cyclin G1, CAT-1, and STAT3) and participating in the process of INH-induced liver injury.

**Electronic supplementary material:**

The online version of this article (10.1186/s40360-018-0201-x) contains supplementary material, which is available to authorized users.

## Background

Isoniazid (INH) is the preferred drug in the treatment of tuberculosis, but the major side-effect in clinical practice is drug-induced liver injury (DILI) [1]. A previous study has shown that the morbidity of hepatotoxicity is 7% from INH and 23% from the administration of anti-tuberculosis drugs rifampicin and pyrazinamide [2]. INH-induced liver injury is related to toxic metabolites [3], immune response [4] and genetic polymorphisms of drug-metabolising enzymes [5]. However, the mechanism of the INH-induced liver injury is not yet fully understood.

MicroRNAs (miRNAs/miRs) are endogenous small non-coding RNAs (19–24 nucleotides) involved in several biological processes, such as lipid metabolism, apoptosis and cancer. Many liver diseases, including fatty liver [6], viral hepatitis [7] and obstructive cholestasis [8], are related to the abnormal changes of miRNAs. The mechanism of miRNA action has been unravelled recently. Most miRNAs can bind with the 3′ untranslated region (UTR) of their mRNA targets for complete or incomplete complementary pairing, resulting in the degradation or translational repression of mRNAs [9]. However, some studies have also shown that miRNAs could bind with other parts of the 3’UTR of their mRNA targets to increase mRNA stability [10, 11]. MiRNAs and their target genes constitute a complex regulatory network, which brings the regulation of mRNA expression and miRNA function to a new level.

More recently, it has been suggested that miRNAs are involved in the incidence and progression of DILI. MiR-122 is a highly abundant and liver-specific miRNA that accounts for 72% of the total liver miRNA population [12]. There was evidence of high miR-122 expression levels in plasma after acetaminophen (APAP) treatment, but their expression levels decreased in the liver [13]. One study also demonstrated that inflammatory miR-125b is dysregulated in APAP-induced liver injury, and this miRNA could potentially represent a biomarker of DILI. Another inflammatory miR-106b has been shown to be associated with halothane-induced liver injury [14]. Although this evidence indicates that these three miRNAs are involved in the occurrence and development of DILI, their detailed effects on INH-induced liver injury remain unknown.

Epigenetic modification, especially DNA methylation, may also influence miRNA genes in regulating miRNA expression. DNA methylation occurs at the C-5 position of the cytosine in CpG dinucleotide sequences, which are mainly concentrated in regions known as CpG islands. Methylation in CpG islands within gene promoters strongly correlates with gene expression [15]. For instance, lower expression levels of miR-200b, miR-152 and miR-10a were associated with increased DNA methylation in bladder cancer [16], and miR-10a was silenced by aberrant DNA methylation in gastric cancer [17]. Because different diseases have unique characteristics, the methylation statuses of different diseases have commonness and personality characteristics. Unfortunately, research on CpG island methylation status of miRNA genes in INH-induced liver injury remains lacking.

Therefore, we first analysed the expression levels of hepatic miR-122, miR-125b and miR-106b after INH administration and explored their correlation with INH-induced liver injury. Following this, we measured the methylation levels of these miRNAs after INH administration and tested whether the methylation levels correlated with their expression levels. Finally, we detected the expression levels of their target genes cell cycle protein G1 (Cyclin G1), cationic amino acid transporter-1 (CAT-1) and signal transducer and activator of transcription 3 (STAT3)) and explored the possible regulation mechanisms of miR-122, miR-125b and miR-106b during INH-induced liver injury.

## Methods

### Animals

Male Sprague-Dawley rats, aged 8–9 weeks old, were purchased from Hua-Fu-Kang Animal Company (Beijing, China). All rats were maintained at 22–23 °C under 65%–69% relative humidity and a 12 h/12 h light/dark cycle with free access to food and water. All rats were acclimatised for one week before the experiment. The experimental protocol was approved by the Institutional Animal Care Committee of North China University of Science and Technology (Permit Number: 14–016).

### Experimental design

After acclimatisation for one week, a total of 56 rats were divided into a control group (*n* = 8) and experimental groups (*n* = 48). The experimental groups were subdivided to 3-, 7-, 10-, 14-, 21- and 28-day groups, which were given INH orally at 50 mg·kg^− 1^·d^− 1^ for each time point. Rats were fasted overnight and sacrificed the following morning by light ether anaesthesia. Plasma and liver tissue samples were collected at the time of animal sacrifice. Blood was drawn by cardiac puncture and collected into silicon disposable glass tubes with ethylenediaminetetraacetic acid (EDTA) as an anticoagulant. Tubes were centrifuged at 4000 g for 15 min at 4 °C. Plasma samples were stored at − 80 °C for later determination of alanine aminotransferase (ALT) and aspartate aminotransferase (AST) activities. Liver tissues were excised immediately and washed with an ice-cold physiologic saline solution (0.9%, *w*/*v*) and blotted dry with filter paper. Liver tissues were split equally into two portions. A portion of liver tissue was fixed in phosphate-buffered formalin and used for histological analysis, while another portion of the liver tissue was stored under refrigeration for subsequent RNA/DNA extraction and polymerase chain reaction (PCR) analysis.

### Histological examination and liver injury scoring

Rat liver specimens, fixed in 10% formaldehyde, went through conventional dehydration, wax-dipping, embedding and slicing. The slices were stained with haematoxylin and eosin (HE) for histopathological examination and scoring. Pathological changes were observed in an optical microscope under 20× magnification [18].

### Biochemical assays

Plasma ALT and AST levels were assessed using an automatic biochemical analyzer (Hitachi, Japan).

### Quantitative real-time PCR (qRT-PCR)

Expression levels of the selected miRNAs (U6, miR-122, miR-125b and miR-106b) and selected mRNAs (β-actin, Cyclin G1, CAT-1, mitogen-activated protein kinase 14 MAPK14, STAT3, RAR-related orphan receptor gamma (RORγt), IL-17, IL-6, TNF-α, CXCL1 and MIP-2) were quantified using real-time RT-PCR analysis. Table 1 lists the primers used in this study. According to the manufacturer’s instructions, rat liver RNA was isolated using TRI reagent (Invitrogen, Carlsbad, CA, USA) and was reverse-transcribed into cDNA. Reverse transcription and real-time RT-PCR were performed as previously described [19]. Relative differences in expression between groups were indicated in terms of cycle time (*Ct*) values, which were generated under the ABI StepOne™ Real-Time PCR System (Applied Biosystems, Carlsbad, CA). Calculations were measured using the 2^−ΔΔCt^ method.

**Table 1:** Sequences of primers used for real-time RT-PCR analyses

Primer Name	Primer sequence
U6	RT: 5′-AACGCTTCACGAATTTGCGTG-3′
	F: 5′-GCTCGCTTCGGCAGCACA-3′
	R: 5′-GAGGTATTCGCACCAGAGGA-3′
miR-122	RT: 5′-GTCGTATCCAGTGCAGGGTCCGAG
	GTATTCGCACTGGATACGACCAAACA-3′
	F: 5′-GGAAAATCGCCATAGCCAGG-3′
	R: 5′-AGATCAGGGTGGCCCCATTT-3′
miR-125b	RT: 5′-GTCGTATCCAGTGCAGGGTCCG
	AGGTATTCGCACTGGAACGACTTCACAA-3′
	F: 5′-CATGGCACTTCCAAGGTTGC-3′
	R: 5′-GCAGACTGACAGACCACACA-3′
miR-106b	RT: 5′-GTCGTATCCAGTGCAGGGTCCGA
	GGTATTCGCACTGGATACGACATCTGC-3′
	F: 5′-CGCCCAGGAAAACATCAAGC-3′
	R: 5′-GGAACTGGCTTTGTTCTGCG-3′
β-actin	F: 5′-GTGGACTAGCAAGCAGGAGT-3′
	R: 5′-CGCAGCTCAGTAACAGTCCG-3′
Cyclin G1	F: 5′-CTGCACGACAACTGAAGCAC-3′
	R: 5′-CTGCGGTACACAGTGAATGC-3′
CAT-1	F: 5′-GCTCCGCAATCCTACACCAT-3′
	R: 5′-GTGGTCAGGACATCGGGTTT-3′
MAPK14	F: 5′-TGCCGTCTCCTTAGGGATGT-3′
	R: 5′-CGCGCCCTTCTCTCCTTTTA-3′
STAT3	F: 5′-TCTGTGTGACACCAACGACC-3′
	R: 5′-AGGCGGACAGAACATAGGTG-3′
RORγt	F: 5′-ACTGACGGCCAGCTTACTCT-3′
	R: 5′-CTGGCACGTCTCTCGGTAG-3′
IL-6	F: 5′-ACAGCGATGATGCACTGTCA-3′
	R: 5′-AGCACACTAGGTTTGCCGAG-3′
TNFα	F: 5′-CTCAAGCCCTGGTATGAGCC-3′
	R: 5′-GGCTGGGTAGAGAACGGATG-3′
IL-17	F: 5′-TCAACCGTTCCACTTCACCC-3′
	R: 5′-CTCCACCCGGAAAGTGAAGG-3′
CXCL1	F: 5′-ACTCAAGAATGGTCGCGAGG-3′
	R: 5′-TTCACCAGACAGACGCCATC-3′
MIP-2	F: 5′-ACCATCAGGGTACAGGGGTT-3′
	R: 5′-CACCGTCAAGCTCTGGATGT-3′

*RT* Reverse-transcription primer, *R* Reverse primer, *F* Forward primer, *Cyclin G1* Cell cycle protein G1, *CAT-1* Cationic amino acid transporter-1, *MAPK14* Mitogen activated protein kinase 14, *STAT3* Signal Transducer and Activator of Transcription 3, *RORγt* Retinoid-related orphan receptorγt, *IL-17* Interleukin-17, *IL-6* Interleukin-6, *TNFα* Tumor necrosis factor alpha, *CXCL1* Cytokineinduced neutrophil chemoattractant 1, *MIP-2* Crophage Inflammatory Protein 2

### Quantitative methylation-specific-PCR (qMSP)

Genomic DNA was extracted from the liver tissue of rats using a DNA Extraction Kit (Aidlab, BJ, China). Extracted DNA was treated with sodium bisulphite using the EZ DNA Methylation-Gold kit (ZYMO Research Corporation, Irvine, CA, USA) according to the manufacturer’s protocols. The CpG island methylation of miR-122, miR-125b and miR-106b was analysed using SYBR Green-based quantitative methylation-specific PCR (qMSP). The UCSC Genome Browser (http://genome.ucsc.edu/) was used to obtain a 2000 bp promoter sequence in the upstream of miR-122, miR-125b and miR-106b genes. Then, we copied the 5′ sequence and fed it into MethPrimer (http://www.urogene.org/methprimer) to predict CpG islands. PCR primers were also designed using the online bioinformatics tool MethPrimer (www.urogene.org/methprimer), which are shown in Table 2. The PCR mixture (20 μL) contained a bisulphite-treated DNA template (2 μL), 2× Power SYBR Green PCR Master Mix (10 μL, Invitrogen, USA) and the forward (1 μL) and reverse (1 μL) primers. PCR conditions included initial incubation at 95 °C for 10 min, 45 cycles of 95 °C for 15 s and 45 cycles of annealing at the temperatures specified in Table 2 for 60 s. PCR products were analysed in 2% agarose gel, stained with ethidium bromide and visualised under ultraviolet (UV) illumination. The CpG methylation percentage in a sample was estimated as previously described [20].

$$ \mathrm{Methylation}\ \mathrm{rate}\left(\%\right)=\frac{M}{M+U}\times 100\%=\frac{1}{1+\frac{U}{M}}\times 100\%=\frac{1}{1+{2}^{-\Delta Ct}}\times 100\% $$

where *M* is the copy number of methylated miR-122, miR-125b and miR-106b; *U* is the copy number of unmethylated miR-122, miR-125b and miR-106b, and *ΔCt* = *Ct*_U_ − *Ct*_M_.

**Table 2:** Primers sequences used in direct qMSP

MiRNA gene	Methylated primers	Unmethylated primers	Temperature (°C)		Amplicon size (bp)	
			M	U	M	U
miRNA-122	F:5′-TTGTTTTGAAAATTATTTTTTTGTTC-3′	F:5′-TTTTGAAAATTATTTTTTTGTTTGA-3′	57.2	57.3	122	120
	R:5′-CATCTACTCACCTAATCCACGAT-3′	R:5′-CCATCTACTCACCTAATCCACAAT-3′	56.5	58.5		
miRNA-125b	F:5′-CGGTTAAAGTATAAATTATAGAGTTACGG-3′	F:5′-TGGTTAAAGTATAAATTATAGAGTTATGG-3′	57.8	57.3	181	179
	R:5′-ACTAACTACAAAACTTCCAAAAACG-3′	R:5′-TAACTACAAAACTTCCAAAAACACC-3′	54.7	56.9		
miRNA-106b	F:5’-TCGGAAATTTATTTGGAAGTTTATC-3’	F:5′-TGGAAATTTATTTGGAAGTTTATTG-3’	59.2	53.9	105	105
	R:5’-GACAATCTACTTCAACTCCTCGAC-3’	R:5’-CAACAATCTACTTCAACTCCTCAAC-3’	57.2	57.7		

### Enzyme-linked immunosorbent assays (ELISA)

Protein contents of Cyclin G1, CAT-1, (MAPK14, p38a), STAT3, IL-17, IL-6, tumor necrosis factor (TNF)-α, chemokine ligand 1(CXCL1), and MIP-2 (MIP-2) in the livers were measured with ELISA Kits (Beijing, Winter Song Boye Biotechnology Co., Ltd., BJ, Beijing, China) according to the manufacturer’s protocols. Intra- and interassay CVs were both less than 15%.

### Statistical analysis

Statistical analysis was performed using SPSS 17.0 software (SPSS, Inc., Chicago, IL, USA). Data were expressed as mean ± SD. Normality and homogeneity-of-variance tests were sequentially conducted on the data. Comparison of variance among groups was performed using one-way analysis of variance (ANOVA). The Student-Newman-Keuls method was used to analyse the differences among the groups. Pearson correlation analyses were used to determine the degree of correlation between different parameters. Differences were considered statistically significant at *p* < 0.05.

## Results

### INH administration caused extensive hepatic damage

**Fig. 1:**
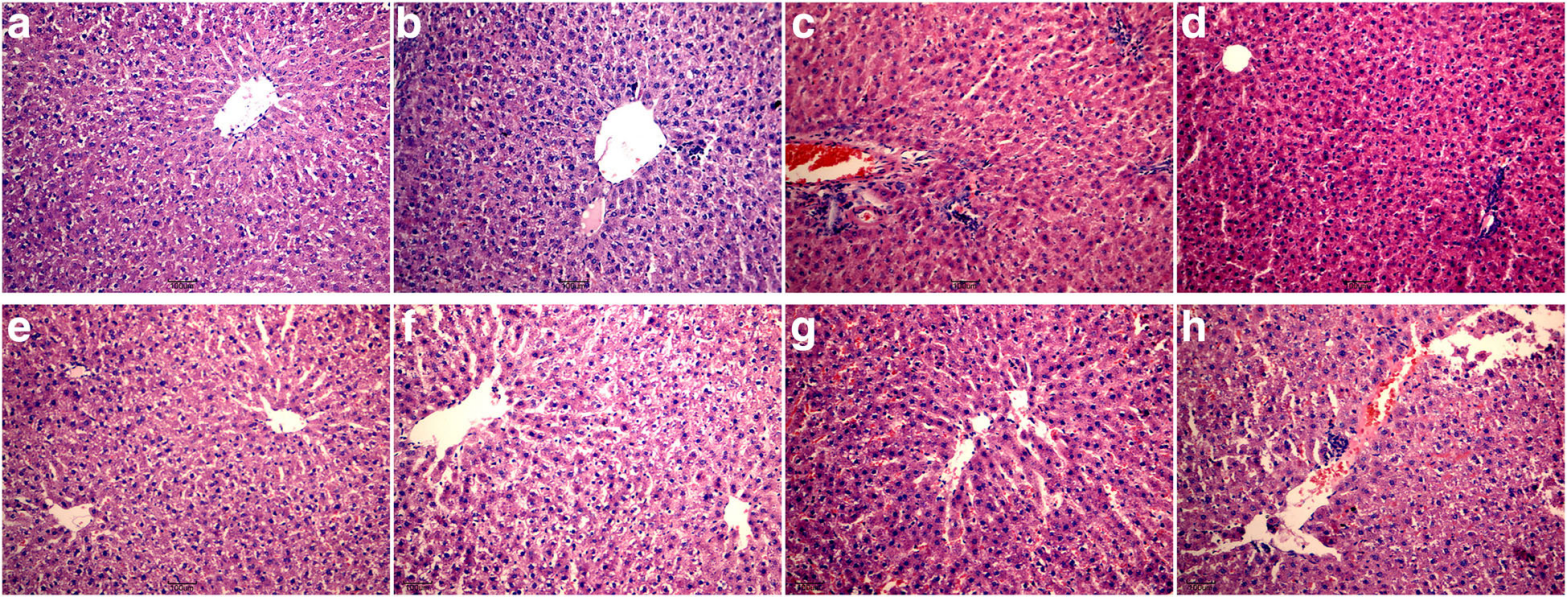
Effect of INH on liver histopathological changes in rats. Rats were administered with 55 mg·kg^− 1^ d^− 1^ INH. At 3, 7, 10, 14, 21 and 28 d after administration, the livers were collected to observe for histopathological changes in HE staining of livers (original magnification, × 20). Representative microphotographs taken from the control (**a**) group or INH-treated for 3 (**b**), 7 (**c**), 10 (**d**), 14 (**e**), 21 (**f**), 28 (**g**) and 28 (**h**) days groups, respectively

Liver histopathology, histological scoring, and ALT and AST activities in serum were evaluated to show the effect of INH on liver injury. Histopathological analysis of liver sections obtained from control rats showed normal architecture of the liver. The structure of liver lobules was intact in the normal control group (Fig. 1a). INH treatment in rats changed the function and structure of liver. The difference in the pathological changes between INH-treated 3-day and 7-day groups was not significant, although a small amount of inflammatory cell infiltration was present (Fig. 1b, c). After 10-day treatment with INH, the hepatocytes were swollen, and necrosis was occasionally visible (Fig. 1d). Ballooning degeneration of liver cells and diffusion were observable at 14 d (Fig. 1e). The liver cells had a more visibly dissolved putrescent state at 21 d (Fig. 1f). The structure of the hepatic cord was heavily destroyed, and many large hepatic cells had necrotised at 28 d (Fig. 1g, h).

**Fig. 2:**
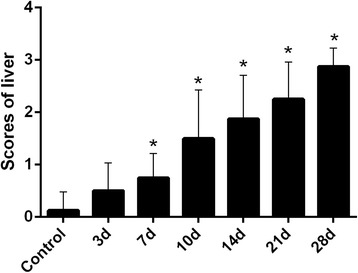
Quantification of histological scoring. Liver pathology scores after 7-, 10-, 14-, 21- and 28-day INH administration were significantly higher compared with that in the normal control group (*p < 0.05 versus control group)

In comparison with the control group, all liver pathology scores dramatically increased at 7, 10, 14, 21 and 28 days after INH administration (Fig. 2 and see Additional file 1, *p* < 0.05 versus control). This result indicates worsening liver damage.

**Fig. 3:**
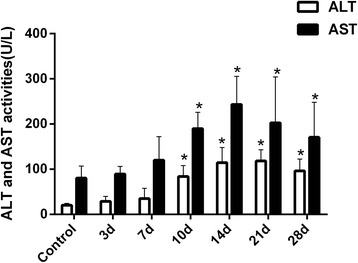
Serum ALT and AST levels of different groups. ALT and AST were significantly higher after 10-day INH administration than the control group (*p < 0.05 versus control group)

ALT and AST are the most sensitive liver enzymes and were widely used as indicators of liver injury. As shown in Fig. 3, the activities of serum AST and ALT after INH administration were significantly higher in the experimental group (Fig. 3 and see Additional file 2, *p* < 0.05 versus control) than in the control group. Both ALT and AST significantly increased after administering INH for 10 days, which proved the occurrence of liver injury.

### Involvement of IL-6 and TNF-α in the pathogenesis of INH-induced liver injury

**Fig. 4:**
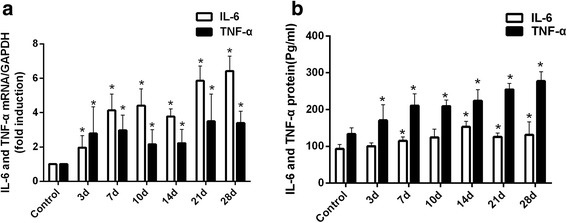
MRNA and protein expression levels of IL-6 and TNF-α in the liver tissue of the different groups. (**a**) IL-6 and TNF-α mRNA expression levels significantly increased in the livers of INH-administered rats compared with the controls. (**b**) IL-6 and TNF-α protein expression levels were significantly higher in the livers of INH-administered rats compared with the controls (*p < 0.05 versus control group)

To explore the involvement of inflammation-related factors in INH-induced liver injury, the hepatic mRNA and protein expression levels of the cytokines, including IL-6 and TNF-α were measured. INH-administered rats showed increased mRNA and protein levels of IL-6 and TNF-α compared to the control rats (Figs. 4a, b and see Additional file 3, *p* < 0.05 versus control). These results suggested that inflammation response participated in INH-induced liver injury.

### Downregulation of miRNA levels were associated with INH-induced liver injury

**Fig. 5:**
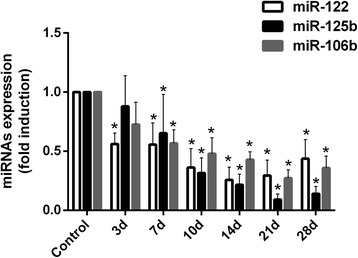
Expression levels of miR-122, miR-125b and miR-106b in the liver tissue of different groups. Hepatic miR-122, miR-125b and miR-106b expression levels dramatically decreased after INH administration for 3, 7 and 7 days (*p < 0.05 versus control group)

To evaluate the relationship of miR-122, miR-125b and miR-106b expression levels with INH-induced liver injury, we examined the expression levels of miR-122, miR-125b and miR-106b using RT-PCR. Compared with the control group, all hepatic miR-122, miR-125b and miR-106b levels dramatically decreased after 3-, 7-, and 7-day INH administration, respectively (Fig. 5 and see Additional file 4, *p* < 0.05 versus control). Moreover, expression levels of miR-122, miR-125b and miR-106b reached a nadir after 14-, 21-, and 21-day administration. These results occurred earlier than the changes of the serum ALT and AST, and histological examination.

The analysis of Pearson correlation coefficients demonstrated that the expression levels of miR-122, miR-125b and miR-106b were negatively correlated with liver scores (*r* = − 0.591, − 0.654 and − 0.701, *p* < 0.001, see Additional file 5: Figure S3) and serum ALT and AST activities (ALT, *r* = − 0.672, − 0.771 and − 0.695, *p* < 0.001, see Additional file 5: Figure S1; AST, *r* = − 0.462, − 0.584 and − 0.606, *p* < 0.001, see Additional file 5: Figure S2). These results suggested that miR-122, miR-125b and miR-106b participated during the early phases of INH-induced liver injury.

### Downregulation of MiRNAs were due to the Hypermethylation of MiRNAs gene promoter region in INH-induced liver injury

**Fig. 6:**
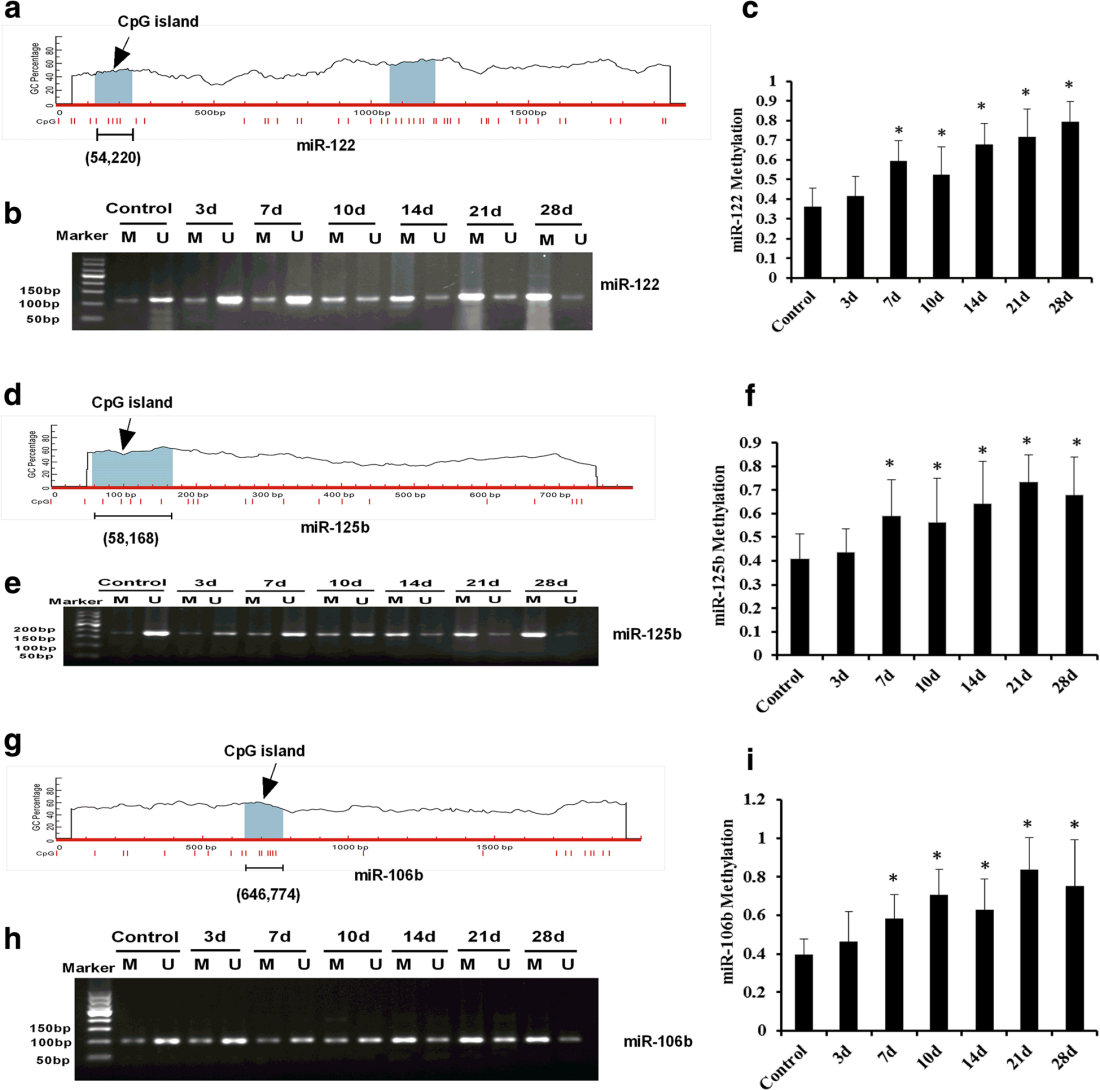
MiR-122, miR-125b and miR-106b were epigenetically silenced in INH-induced liver injury. (**a**), (**d**) and (**g**) represent the CpG islands of miR-122, miR-125b and miR-106b that were predicted by MethPrimer, respectively. Agarose gel electrophoresis of the PCR products of gene promoter methylation of (**b**), miR-122, (**e**) miR-125b and (**h**) miR-106b. Marker: DNA ladder 50 bp, M: Methylation, U: Unmethylation. DNA methylation at particular CG dinucleotides within the miR-122 gene promoter (**c**), miR-125b gene promoter (**f**) and miR-106b gene promoter (**i**) in liver tissues from INH-administered rats was determined by qMSP. Methylated levels of miR-122, miR-125b and miR-106b in INH-administered rat liver tissues were significantly higher than those in the control rats (*p < 0.05 versus control group)

Previous studies have demonstrated that hypermethylation of CpG islands in the promoter region of miRNA genes is one of the most important mechanisms that leads to the downregulation of miRNA expression [21]. The analysis of the promoter region revealed that miR-122, miR-125b and miR-106b had CpG islands within the 2000 bp upstream of the transcriptional start site (Fig. 6a–e), providing the structural basis of DNA methylation. Thus, we hypothesised that hypermethylation is responsible for the downregulation of these three miRNAs. To test this hypothesis, we performed quantitative methylation-specific PCR (qMSP) analysis to detect the methylation levels of the promoter regions of these three miRNAs. Results demonstrated that the methylation levels of miR-122, miR-125b and miR-106b in rat liver tissues treated with INH were significantly higher than those in the control group (Figs. 6c–i and see Additional file 6, *p* < 0.05 versus control). In addition, hepatic miR-122, miR-125b and miR-106b methylation levels significantly increased at 7 days after INH administration. Products from methylated and unmethylated miR-122 primers were 122 and 120 bp in size, respectively (Fig. 6b). Products of the methylated and unmethylated miR-125b primers were 181 and 179 bp in size, respectively (Fig. 6e). Both products of the methylated and unmethylated miR-106b primers were 105 bp in size (Fig. 6h).

We also analysed the correlation between methylation levels and the gene expression of miR-122, miR-125b and miR-106b. The results showed the negative correlation between methylation and expression levels of miR-122, miR-125b and miR-106b (miRNA-122, *r* = − 0.587, *p* < 0.001; miRNA-125b, *r* = − 0.536, *p* < 0.001; miRNA-106b, *r* = − 0.568, *p* < 0.001, see Additional file 5: Figure S4). Thus, the correlation between the expression levels and methylation of CpG islands of the miR-122, miR-125b and miR-106b genes have been revealed, indicating the possibility of epigenetic regulation of these genes during INH-induced liver injury.

### MiRNA-122 regulated Cyclin G1 and CAT-1 in INH-induced liver injury

Our results indicated that the expression level of miR-122 decreased at 3 days after INH administration and declined to a minimum at 14 days, before rising rapidly (Fig. 5). This phenomenon raised the question of what the role miR-122 played during INH-induced liver injury. The biological target genes of miR-122 should be taken into consideration. We search for putative miR-122 target genes that may participate in the regulation of liver injury. Previous studies identified cell cycle protein G1 (Cyclin G1) and the cationic amino acid transporter-1 (CAT-1) were targets of miR-122 [22, 23]. Cyclin G1 and CAT-1 played a crucial role in cell survival and proliferation in the carbon tetrachloride- and thioacetamide-induced liver injury models [24]; however, their role in INH-induced liver injury models has yet to be evaluated under the rationale for testing Cyclin G1 and CAT-1 expression in our INH-induced liver injury model.

**Fig. 7:**
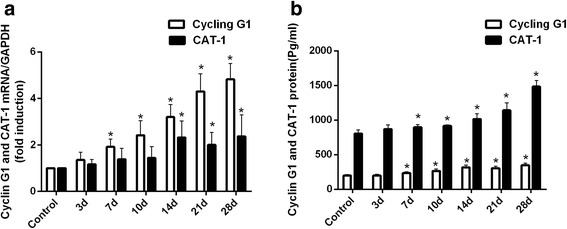
mRNA and protein expression levels of Cyclin G1 and CAT-1 in the liver tissue of the different groups. (**a**) Cyclin G1 and CAT-1 mRNA expression levels were significantly higher in the livers of INH-administered rats compared with the control group. (**b**) Cyclin G1 and CAT-1 protein expression levels were significantly higher in the livers of INH-administered rats compared with the controls (*p < 0.05 versus control group)

Along with lower miR-122 (Fig. 5), we found higher levels of Cyclin G1 and CAT-1 mRNAs in the livers of the experimental group rats compared with the control (Fig. 7a). All Cyclin G1 and CAT-1 mRNA levels significantly increased at 7 days after the INH treatment (Fig. 7a and see Additional file 7, *p* < 0.05 versus control).

To investigate the role of Cyclin G1 and CAT-1 in INH-induced liver injury, we measured the Cyclin G1 and CAT-1 protein levels. Expectedly, we found a significant increase of Cyclin G1 and CAT-1 proteins in the livers (Fig. 7b and see Additional file 7, *p* < 0.05 versus control). The higher protein levels might suggest that miRNA-122 could upregulate translation.

Generally, the low expression level of miR-122 and high expression of Cyclin G1 and CAT-1 at both mRNA and protein levels during INH-induced liver injury suggested that miR-122 could upregulate translation.

### MiR-125b and MiR-106b regulated STAT3 in INH-induced liver injury

Previous studies have confirmed that miR-125b and miR-106b were two important indicators that reflect inflammatory response. Our results also confirmed that both miR-125b and miR-106b expression levels dramatically decreased during INH-induced liver injury. The time of significant changes and the changing trends of miR-125b and miR-106b were all consistent. We also found a higher degree of inflammatory cell infiltration in liver histopathology (Fig. 1b) and significantly increased mRNA and protein levels of IL-6 and TNF-α in the livers (Fig. 4a, b) after 3 days of INH administration. Altogether, this discovery raised the question of whether miR-125b and miR-106b were related to the inflammatory immune response in INH-induced liver injury.

**Fig. 8:**
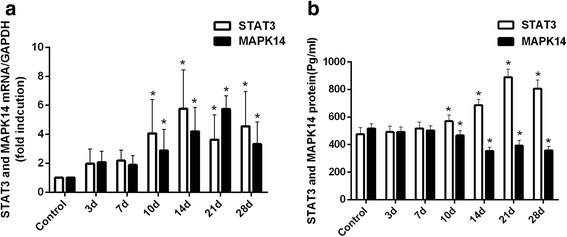
mRNA and protein expression levels of STAT3 and MAPK14 in the liver tissues of different groups. (**a**) STAT3 and MAPK14 mRNA expression levels were significantly higher in the livers of INH-administered rats after 10 days compared with those in the control group. (**b**) STAT3 protein expression levels were significantly higher, but MAPK14 protein levels were lower in the livers of INH-administered rats after 10 days compared with the control (*p < 0.05 versus control group)

First, we searched for putative miR-125b and miR-106b targets that might regulate inflammatory immune response. These gene targets are identified as MAPK14 and STAT3. Previous studies confirmed that MAPK14 and STAT3 were common gene targets of miR-125b and miR-106b [25–27]. Considering the fact that one miRNA can regulate multiple target genes, and several miRNAs can regulate the single gene to exert its effect, the modulation of MAPK14 and STAT3 during INH-induced liver injury may be conceivable. In this study, we found significant elevation of STAT3 in both mRNA and protein levels after administering INH for 10 days (Fig. 8a, b and see Additional file 8, *p* < 0.05 versus control). MAPK14 mRNA levels were higher (Fig. 8a), but the protein levels were lower (Fig. 8b), indicating the absence of correlation between these expression levels. These results suggested the possible activation of STAT3 during INH-induced liver injury.

**Fig. 9:**
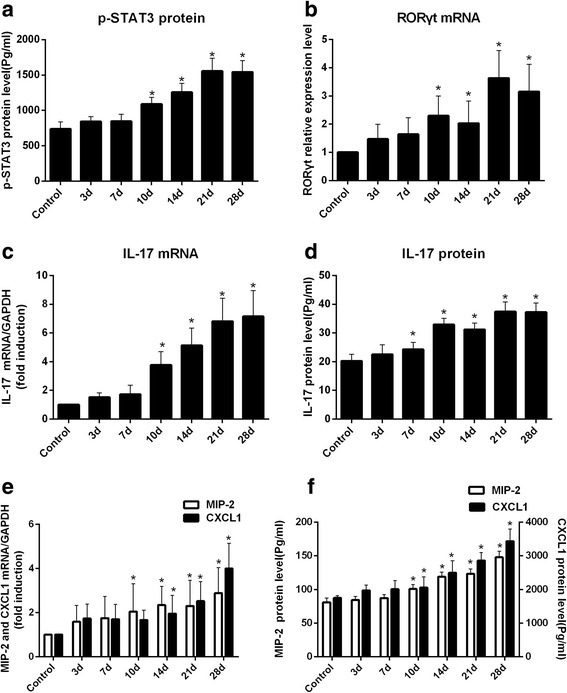
INH administration causes alterations in RORγt (**b**), IL-17 (**c**), MIP-2, and CXCL1 (**e**) mRNA expression levels and p-STAT3 (**a**), IL-17 (**d**), MIP-2 and CXCL1 (**f**) protein levels in the liver

To explore the activation of STAT3, we detected the protein content of its activated form, p-STAT3. We found that the protein content of p-STAT3 was significantly higher in the livers of the experimental group rats after 10-day INH administration than those of the control group (Fig. 9a and see Additional file 8, *p* < 0.05 versus control). The time of significant change in p-STAT3 was consistent with STAT3. These results showed that STAT3 was activated during INH-induced liver injury.

Some studies confirmed that STAT3 further regulated its downstream RORγt gene to induce inflammatory cytokine IL-17 [28]. We detected mRNA levels of RORγt, and found that RORγt mRNA levels significantly increased in the livers of experimental group rats compared with those of the control group after 10-day administration of INH in the former (Fig. 9b and see Additional file 9, *p* < 0.05 versus control). In addition, IL-17, a newly discovered pro-inflammatory cytokine, is highly abundant in drug-induced liver injury [29] and produces the chemokines CXCL1 and MIP-2 [30]. The mRNA and protein levels of IL-17, CXCL1 and MIP-2 also significantly increased in INH-administered rats (Figs. 9c–f and see Additional file 9, *p* < 0.05 versus control). The above results suggested that the aberrantly downregulated miR-125b and miR-106b upregulated the STAT3 expression to stimulate the secretion of inflammatory factors during INH-induced liver injury.

## Discussion

In the present study, the expression levels of hepatic miR-122, miR-106b and miR-125b were downregulated and correlated with liver pathology scores and serum AST and ALT activities, suggesting that the dynamic change in the expression levels of miR-122, miR-106b and miR-125b were related to INH-induced liver injury. Methylated levels of miR-122, miR-106b and miR-125b were upregulated in INH-induced liver injury and correlated with their expression levels. These results suggested that methylation was responsible for the downregulation of miR-122, miR-106b and miR-125b expression during INH-induced liver injury. INH also decreased the expression of miR-122, which corresponded to the increase of two gene targets: Cyclin G1 and CAT-1. Finally, the aberrant downregulation of miR-125b and miR-106b upregulated the STAT3 expression to stimulate the secretion of inflammatory factors during INH-induced liver injury.

MiRNAs have emerged as an important mechanism in drug-induced liver injury. Mitsugi et al. [31] implicated miR-877-5p-induced PEPCK as a trigger involved in the development of trovafloxacin-induced liver injury. Uematsu et al. [32] reported that regulating the expression of SRY-box 4 (SOX4) and lymphoid enhancer-binding factor 1 (LEF1) by miR-29b-1-5p and miR-449a-5p are important in the development of Th2 bias in methimazole-induced liver injury. These studies suggest the importance of miRNAs in the regulation of drug-induced liver injury. Changes in miRNA profiles, including lower miR-122, miR-106b and miR-125b levels, have been reported in animal model studies on drug-induced liver injury. However, the expression of miRNAs in INH-induced liver injury remains largely unexplored. To our knowledge, this study shows for the first time that hepatic miR-122, miR-125b and miR-106b expression levels gradually decreased during a model of INH-induced liver injury. Moreover, we clearly found that the expression of miR-122, miR-125b and miR-106b were significantly lower at different times. The miR-122 expression was significantly lower after 3 days, while both miR-125b and miR-106b significantly decreased after 7 days. The reason for this change might be related to the tissue specificity of miRNA expression. miR-122 was one of the most abundant miRNAs in the liver, accounting for up to 72% of all hepatic miRNAs [12], while the expressions of miR-106b and miR-125b not only occur in the liver, but also in many other tissues, including the uterus, ovaries and lungs. A common characteristic of these miRNAs in the expression was that they initially decreased before gradually increasing through time. The occurrence of drug-induced liver injury can explain this phenomenon. Unlike other organs, the liver has an amazing regenerative ability, as evidenced by its recovery after toxic and drug-induced liver injury [33–35]. We further analysed whether the expression levels of miR-122, miR-106b and miR-125b are correlated with the ongoing liver damage according to liver histopathology and serum ALT and AST activities. miR-122, miR-106b and miR-125b expression levels are correlated with liver pathology scores and AST and ALT levels, supporting the correlation of these three miRNAs with INH-induced liver injury.

Previous studies have shown that aberrant hypermethylation of CpG islands in miRNA promoter sequences occurred in some cancer types, such as gastric, oral and hepatocellular cancer, leading to the downregulation of miRNA expression [36–38]. Another study found that the expression of miR-34a during alcoholic liver injury might be regulated by methylation [39]. These publications demonstrated that methylation of CpG-islands affects miRNA gene expression. Thus, we hypothesise that methylation is responsible for the downregulation of miR-122, miR-125b and miR-106b in INH-induced liver injury. To verify this hypothesis, we used qMSP analysis to detect the methylation level of hepatic miR-122, miR-125b and miR-106b. As a result, the methylation of CpG islands in these three miRNA genes was detected. For the first time, we have detected the frequent methylation of miR-122, miR-125b and miR-106b. In our work, statistically significant correlation was detected between the expression level of miRNA genes and the methylation status of CpG islands of these genes in the liver tissue, indicating the possibility of the epigenetic regulation of these genes during INH-induced liver injury.

To develop a deeper understanding of the molecular mechanism of miRNA genes involved in INH-induced liver injury, mRNA and protein expression of the miRNA gene targets were determined. CAT-1 and Cyclin G1 genes are miR-122 targets. We have revealed that INH decreased the expression of miR-122, which corresponded with the increase of Cyclin G1 and CAT-1. However, we revealed a situation in which the expression level of miR-122 did not continuously decrease after INH administration. Instead, miR-122 levels initially declined before rising. We also found that ALT and AST activities were not always higher after INH administration, as both reached a maximum at 21 and 14 days, respectively, before decreasing. These phenomena can be partially explained by liver regeneration. CAT-1 is responsible for cationic amino acid transport, and this intake stimulates DNA synthesis for cell replication [40]. Cyclin G1, a cell-cycle-regulatory protein, participates in G2/M arrest of cells in response to DNA damage, promotes DNA damage repair, and induces apoptosis [41]. Previous studies have also discovered that Cyclin G1 is important for cell proliferation [42]. Therefore, the reduction of miR-122 levels may regulate the target genes, Cyclin G1 and CAT-1, to activate cell proliferation and to repair the damaged liver tissue caused by INH. However, the specific biological role of Cyclin G1 and CAT-1 in INH-induced liver injury deserves further investigation.

DILI pathogenesis usually involves parent compounds that become hepatotoxic through metabolism, oxidative stress, lipid peroxidation, mitochondrial dysfunction, apoptosis and immune response [43]. A growing body of research suggests that inflammation is an important factor that contributes to DILI. miR-125b and miR-106b are two miRNAs identified to be associated with inflammation. We attempted to elucidate the underlying mechanism of miR-125b and miR-106b involved in INH-induced liver injury. TNF-a and IL-6 are key pro-inflammatory cytokines that initiate the inflammatory response and induce massive hepatocyte apoptosis. Previous studies have demonstrated that the liver can activate Kupffer cells to release inflammatory cytokines (IL-6 and TNF-α) and to aggravate hepatic cell death when stimulated by toxic substances [28, 30]. In the present study, analysis of inflammatory indicators in rats after INH administration showed that TNF-a and IL-6 mRNA and protein levels in the liver tissue of experimental groups were significantly higher than those of the control group. At the same time, a large number of capillaries and inflammatory cell infiltration was also present in liver pathology. Therefore, INH can activate inflammation during liver injury. We also found that STAT3, an important signal transduction pathway, was predicted and experimentally proven as the common gene target of miR-125b and miR-106b [25–27]. STAT3 can be activated in hepatocytes following TNF-α and IL-6 stimulation. STAT3 activation plays an important role in cell survival, differentiation, transformation, apoptosis and inflammation [44]. In this study, we showed that STAT3 in mRNA and protein levels increased, and the protein content of p-STAT3 was significantly higher after INH administration. These results suggested the possible activation of STAT3 during INH-induced liver injury. STAT3 is an important determinant in the differentiation of T-helper 17 (Th17) cells and regulates the transcription of inflammatory genes [45]. Th17 cells mainly secrete pro-inflammatory cytokine IL-17 [46], which stimulate the activation of various pro-inflammatory cytokines and chemokines and participate in drug-induced liver injury [47]. Given this information, we speculate that STAT3 activation induces IL-17 to produce chemokines, such as MIP-2 and CXCL1, and exacerbates INH-induced liver injury. To confirm this hypothesis, we examined the expression of IL-17, MIP-2 and CXCL1 in both mRNA and protein levels after INH treatment and found their enhanced levels under such conditions. Taking these experimental data into consideration, we can conclude that aberrant downregulation of miR-125b and miR-106b can upregulate STAT3 expression to stimulate the secretion of inflammatory factors involved in INH-induced liver injury.

## Conclusion

In this study, lower hepatic levels of miR-122, miR-125b and miR-106b are associated with INH-induced liver injury. CpG island hypermethylation of miR-122, miR-125b and miR-106b genes correlate with their expression levels. Furthermore, the expression level of miR-122 has a causal role in higher levels of Cyclin G1 and CAT-1 mRNA and protein expression. Furthermore, the expression of miR-125b and miR-106b has a causal role in enhanced STAT3 mRNA and protein expression during INH-induced liver injury. Our results suggested that DNA methylation likely regulated the expression of miRNA genes (miR-122, miR-125b and miR-106b), thereby affecting the expression of their target genes (Cyclin G1, CAT-1 and STAT3) and participating in the process of INH-induced liver injury.

## Additional files

Additional file 1: Quantification of histological scoring. Supplementary data and statistical analysis for **Figure S3**. (XLSX 16 kb)

Additional file 2: Serum ALT and AST levels of different groups. Supplementary data and statistical analysis for **Figure S4**. (XLSX 19 kb)

Additional file 3: mRNA and protein expression levels of IL-6 and TNF-α in the liver tissue of the different groups. Supplementary data and statistical analysis for **Figure S5**. (XLSX 27 kb)

Additional file 4: Expression levels of miR-122, miR-125b, and miR-106b in the liver tissue of different groups. Supplementary data and statistical analysis for **Figure S6.** (XLSX 24 kb)

Additional file 5: Figures of pearson correlation analysis. (DOC 917 kb)

Additional file 6: DNA methylation at particular CG dinucleotides within the gene promoter of miR-122, miR-125b, and miR-106b in liver tissues from INH-administered rats . Supplementary data and statistical analysis for **Figure S7.** (XLSX 25 kb)

Additional file 7: mRNA and protein expression levels of Cycling G1 and CAT-1 in the liver tissue of the different groups. Supplementary data and statistical analysis for **Figure S8.** (XLSX 27 kb)

Additional file 8: mRNA and protein expression levels of STAT3 and MAPK14 in the liver tissue of different groups. Supplementary data and statistical analysis for **Figure S9.** (XLSX 28 kb)

Additional file 9: INH administration causes alterations in RORγt, IL-17, MIP-2, and CXCL1 mRNA expression levels and p-STAT3, IL-17, MIP-2, and CXCL1 protein levels in the liver. Supplementary data and statistical analysis for **Figure S10.** (XLSX 51 kb)

## Abbreviations

ALT: Alanine aminotransferase
ANOVA: Analysis of variance
AST: Aspartate aminotransferase
CAT-1: Cationic amino acid transporter-1
CXCL1: Cytokineinduced neutrophil chemoattractant 1
Cyclin G1: Cell cycle protein G1
DILI: Drug-induced liver injury
ELISA: Enzyme Linked Immunoasorbent Assays
F: Forward primer
HE: Hematoxylin and eosin
IL-17: Interleukin-17
IL-6: Interleukin-6
INH: Isoniazid
MAP K14: Mitogen activated protein kinase 14
MIP-2: Crophage inflammatory protein 2
qMSP: Quantitative Methylation-Specific-PCR
qRT-PCR: Quantitative Real-Time Polymerase Chain Reaction
R: Reverse primer
RORγt: Retinoid-related orphan receptorγt
RT: Reverse-transcription primer
SD: Standard deviation
STAT3: Signal Transducer and Activator of Transcription 3
TNF-α: Tumor necrosis factor alpha
