# Supplementary material for: Involvement of methylation of MicroRNA-122, −125b and -106b in regulation of Cyclin G1, CAT-1 and STAT3 target genes in isoniazid-induced liver injury
Source: BMC Pharmacol Toxicol. 2018 Mar 20;19:11. doi: 10.1186/s40360-018-0201-x (PMC5859513; doi:10.1186/s40360-018-0201-x)
Supplement: Supplementary file 5 — Figures of pearson correlation analysis. (DOC 917 kb) [file 40360_2018_201_MOESM5_ESM.doc]

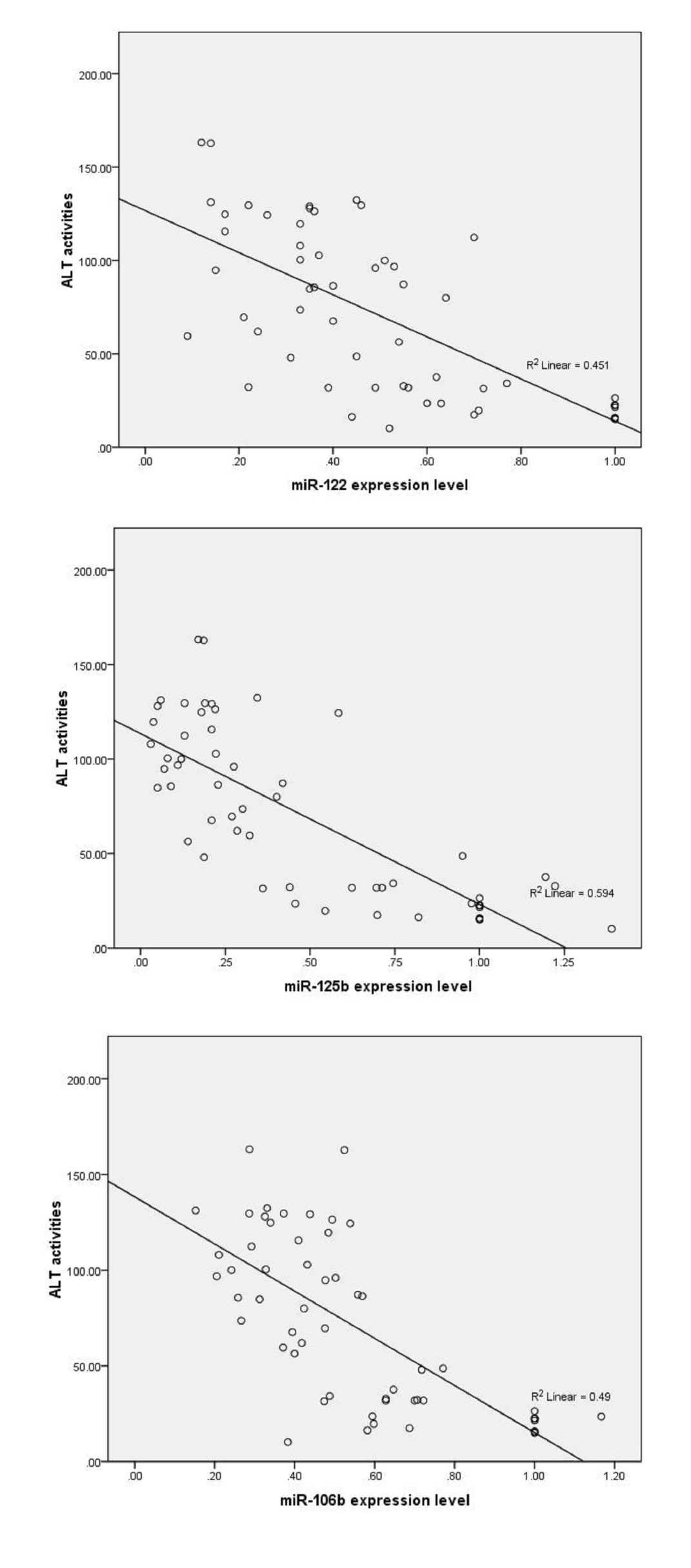


Figure S1 Correlation between relative expression of miR‑122, miR‑125b and miR‑106b and ALT activity (n=8, *r*=coefficient of determination).


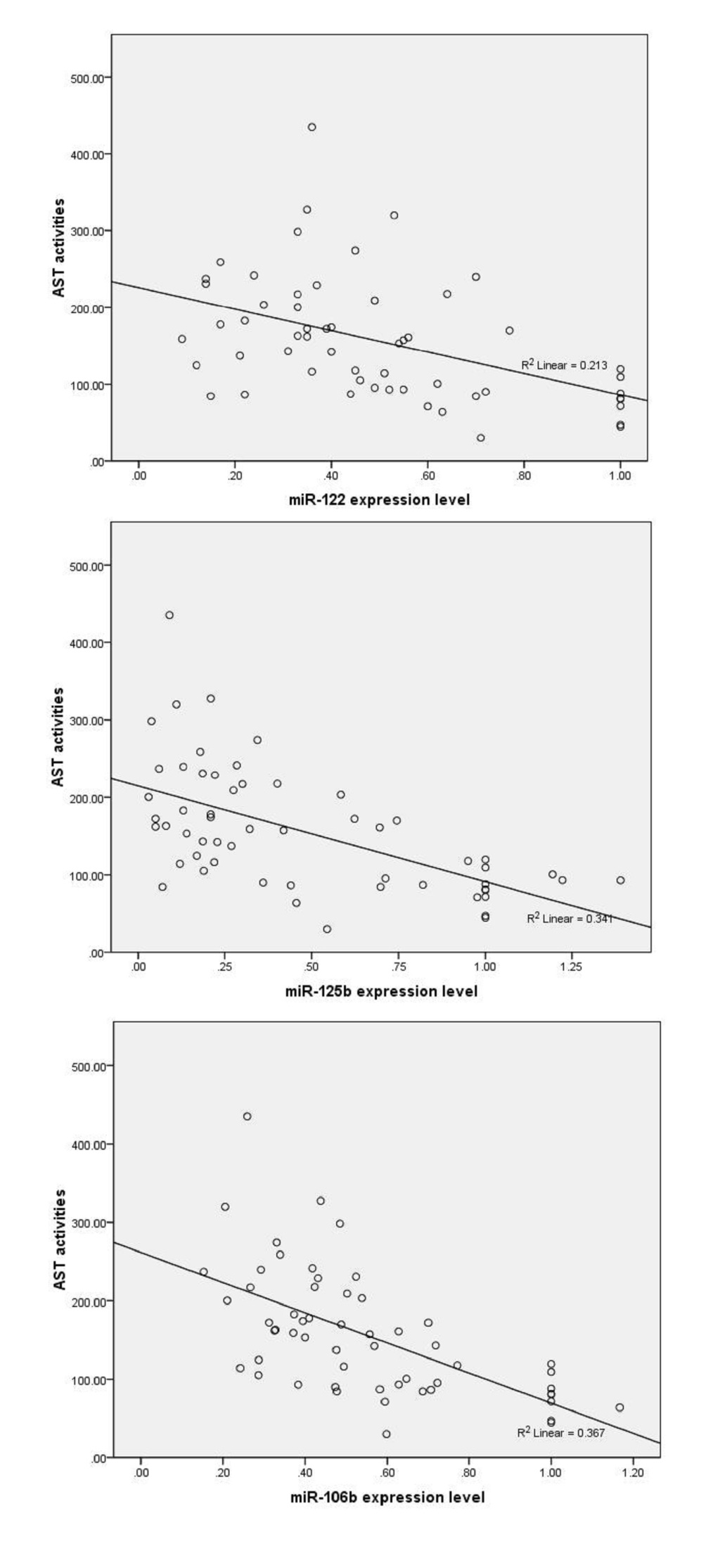


Figure S2 Correlation between relative expression of miR‑122, miR‑125b and miR‑106b and AST activity (n=8, *r*=coefficient of determination).


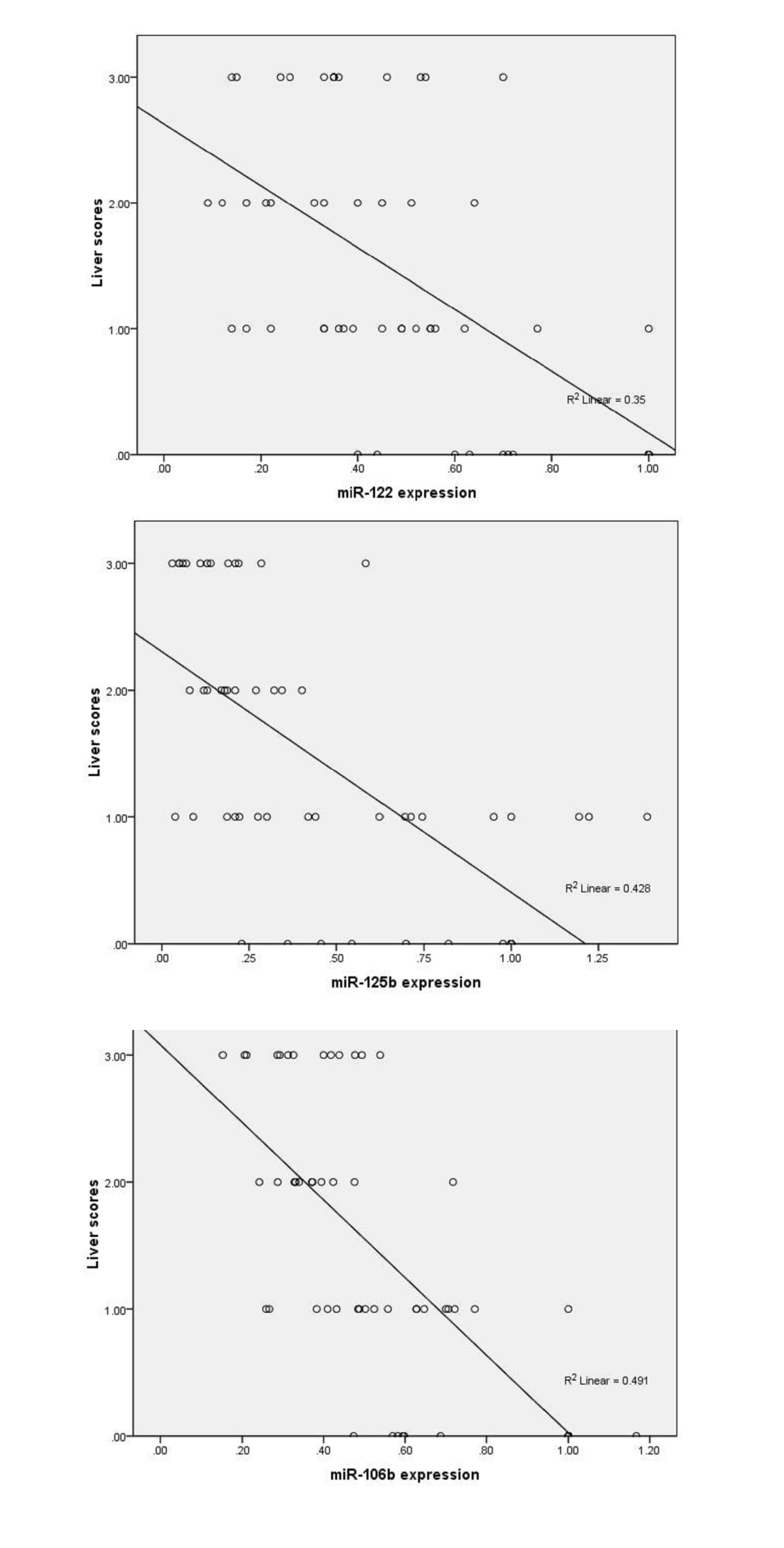


Figure S3 Correlation between relative expression of miR‑122, miR‑125b and miR‑106b and liver scores (n=8, *r*=coefficient of determination).


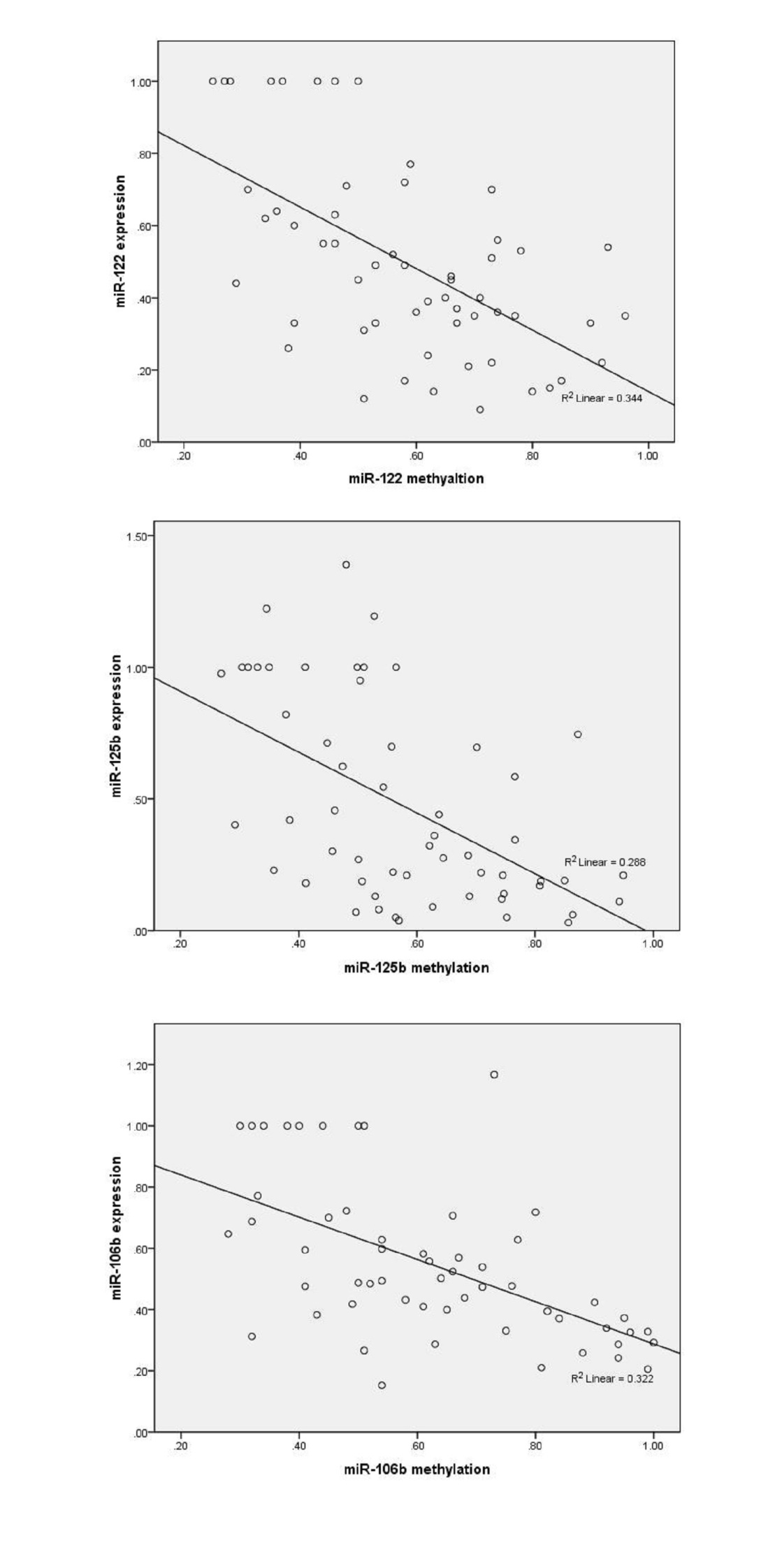


Figure S4 Correlation between the expression and methylation of miR-122, miR-125b and miR-106b (n=8, *r*=coefficient of determination)


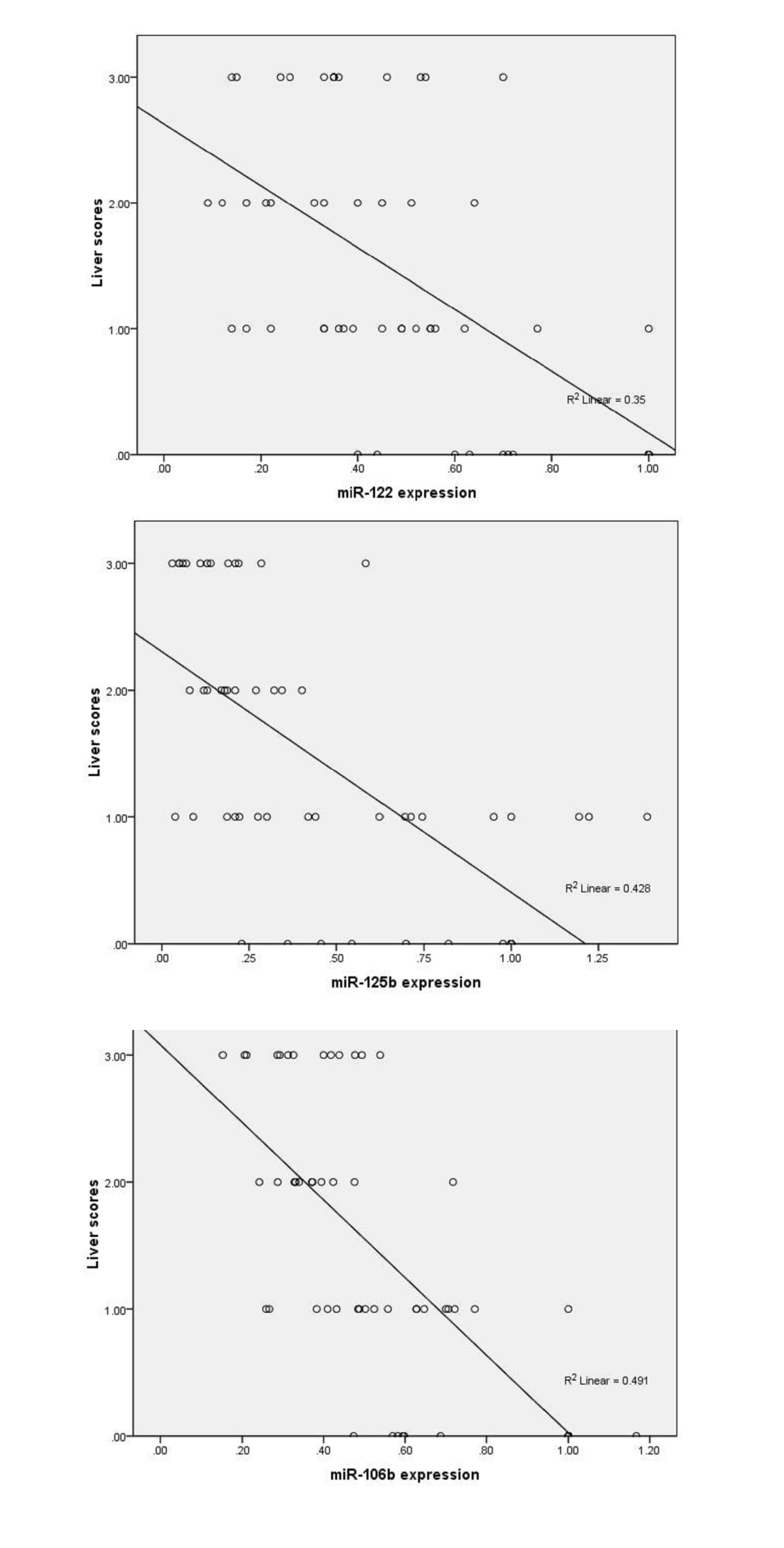


Figure S5 Correlation between the relative expression of miR‑122, miR‑125b and miR‑106b expression and liver scores (n=8, *r*=coefficient of determination)
